# Notes on the Prognosis and Treatment of Pellagra

**Published:** 1909-11

**Authors:** C. H. Lavinder

**Affiliations:** Passed assistant surgeon, United States Public Health and Marine Hospital Service


### Notes on the Prognosis and Treatment of Pellagra.

By C. H. Lavinder, passed assistant surgeon, United States Public Health and Marine Hospital Service.

(*Public Health Reports, September 10, 1909.*)

PROGNOSIS.

IN undertaking any discussion of the prognosis of pellagra as seen in the United States, there are at least two factors which must not be overlooked. The first is that our comparatively brief experience with the disease in this country should make us guarded in our statements, and the other is that a large part of our published experience is based on asylum cases of the disease, which are usually regarded as the most hopeless.

Generally speaking, it may, I think, be safely said that in this country at least the prognosis in all cases is grave as to final and complete recovery The statistics in existence, all founded on asylum cases, and not a very large number at that, will give an average case mortality of about 67 per cent., a state of affairs which, to say the least, is not conducive to optimism.

All American physicians who have had experience seem to regard the outlook in individual cases as one of great gravity.

T. C. Allbutt (Albutt's System of Medicine, Vol. II, 1897) says: "When the disease has recurred for three or four seasons, and especially if the mind be affected, the prognosis is very bad. I gathered from the physicians of Italian lunatic asylums that recovery of patients once arrived at the asylum stage of insanity is almost unknown. Still these are extreme cases; the mentally afflicted in their earlier phases may recover; only too often, however, the advance of death is inexorable." And this, I think, expresses very fairly the view generally entertained in the United States.

This view, however, may be unduly pessimistic. Lombroso states (Trattato profilattico e clinico della pellagra, Turin, 1892) that in 1883 there were treated in 866 Italian civil hospitals 6,025 pellagrins, of whom 923 died; in 1884 there were treated in 993 hospitals 6,944, of whom 780 died, thus giving, on a large experience, an average case mortality very close to 13 per cent. Wollenberg (Public Health Reports, July 23, 1909) reports from credible sources a total of 55,029 cases of pellagra in Italy in 1905, with a total mortality of 2,359, which is a little over 4 per cent.

Babes and Sion (Spec. Path. u Therap. Nothnagel, Band, XXIV), in dealing with nonasylum cases, state that with proper treatment complete cure of psychic as well as motor changes may result. They also state that the disease can be strikingly improved or cured not only in early but in more advanced cases, though the prognosis is far better in early cases.

It is probably safe to assert that as a rule the earlier the diagnosis is made and treatment begun the better the prognosis. The diagnostician then should learn to profit by a similar experience in tuberculosis in which the situation is in some respects analogous.

Pellagra, like tuberculosis, is a very chronic condition with, in non-asylum cases, perhaps just as hopeful an outlook. We should profit by experience, learn to make diagnosis, and institute proper treatment in the early stages of the disease and hopefully counsel our pellagra patients as we do our tubercle cases. Pellagra is said in Italy to last as long as twenty-five years; and Babcock in South Carolina has seen cases of eight and twelve years' standing who were still in very good physical condition and showed improvement under treatment, if not recovery. It is true, however, that pellagra is variable in its manifestations, and acute accidents and grave complications are frequent.

The chronic type of the disease, without mental involvement, gives the most hopeful outlook. Acute manifestations must be viewed with the utmost gravity.

Pellagra is a disease of little fever, and it is, I think, the general opinion that fever, particularly if high or constant, must be regarded as a danger signal. The state of the erythema is generally thought to bear no relation to the gravity of the constitutional disturbances. It has been my experience, however, that moist, extensive erythemas are frequently accompanied by grave constitutional changes. Mental involvement, as stated, adds to the seriousness of the case; and such nervous disturbances as subsultus, marked tremor, retraction of the head, can, as in other affections, be interpreted as an index of severe intoxication. In mental cases periods of excitement are not rare, and they do much to help exhaust the patient. Severe recrudescence of the acute phenomena sometimes occur during the same season after the patient seemed to be on the road to recovery. Steadily progressing emaciation, especially if accompanied by an inveterate diarrhea, which is usual, very often ends fatally.

Certain complications are of great importance in prognosis, e. g., malaria, intestinal parasites, marked nephritis, acute bronchitis, pneumonia, decubitus gangrene (which is often difficult to avoid), possibly tubercle and at times hyperpyrexia, due probably to a sudden overwhelming dose of toxic material. Then, of course, if a patient is carried through his summer manifestations safely one year, a reappearance of acute manifestations the next year must be watched for, more especially if anything should intervene to lower the general resistance, such as acute illness, childbirth, and the like.

PROPHYLAXIS.

In any discussion of treatment we must first of course recognise the paramount importance of prophylaxis. Whatever views one may entertain as to the cause of the disease there seems to be an almost universal belief that there is some definite etiological relation between Indian corn and pellagra. In dealing with a disease of such gravity, a belief so universal as this can not be discarded except in the face of conclusive proof to the contrary. There are also the best of reasons for thinking that poverty, especially abject poverty, and all that is implied in that term—poor and insufficient food, bad housing, unhygienic surroundings, mental depression, lowered physical resistance, and often alcoholism—have a greater effect than usual in predisposing to pellagra; and predisposition in this disease is generally admitted to be a factor of the greatest importance.

What shall be done then in the way of prophylactic measures? It is evident of course that as far as possible distress, poverty, and unhygienic surroundings should be relieved, alcohol interdicted, and the individual, as well as the community, placed under the best possible circumstances. This is nothing new of course and will receive the assent of all, but in Italy such unique attempts at general preventive measures have been adopted along this line as to give this statement a new meaning. Various establishments for the prophylaxis of the disease have been originated and are said to have been of aid in the production of hopeful results, such as the *pellagrosari, forni economici, forni rurali, cucine economice, locande sanitarie,* all of which are devoted to feeding, treating, and educating the unfortunate sufferers.

So far as a dietary containing corn is concerned, there is abundant evidence that good corn is not only a wholesome but a harmless food, and not a few writers have pointed out the folly of those who counsel the total rejection of so valuable a cereal. At the same time, entirely wholesome corn is not always easily differentiated from harmful corn. In the light of our present knowledge, therefore, maize should be admitted, it seems to me, into the dietary of certain institutions, like insane asylums, with the utmost caution. As for the use of corn or its products elsewhere or in one's individual diet, that is a matter which is as yet, to some extent, *sub judice,* and must for the time perhaps be left to individual judgment.

TREATMENT.

Regarding the medical treatment of the disease, Sir Henry Holland wrote, in 1817 (Medico-Chirurgical Transactions, London, 1820): "In short, it appears certain that mere medicine has done very little for the relief of pellagra; and Strambio, a man with large experience in asylums, frankly confesses that he never saw a case distinctly cured by the remedies that were employed."

Certainly we must admit, at the outset, that we have no specific for the disease; but since Holland's time Lombroso's magnificent work on pellagra has been done, and while by some he may be considered as too optimistic on treatment, his enormous experience certainly entitles his views to the greatest attention and respect. He says, after discussing the use of arsenic in the treatment of pellagra, that the therapy, which was at first desperate and could be summed up in baths barren of result, can now be undertaken more confidently and rationally, as the treatment of a chronic intoxication analogous to alcoholism or morphinism and curable by antidotes when the use of the toxic material has been suspended. (The antidotes referred to are arsenic and chloride of soda, concerning which more has been said elsewhere.)

Lombroso's teaching on therapy has had such a profound effect that it may be wise to give briefly some account of his views.

He recommends as a rule a liberal diet, including meats especially, but points out that this alone is insufficient. He also remarks that in well-nourished pellagrins this is of course not so much indicated, and adds that such cases are rebellious to treatment. He speaks of baths and cold douches, which he thinks benefit especially paretic states, the skin manifestations and the painful burning sensations so common in pellagrins; and further says that, while they do not cure, they at least prolong existence or render it more tolerable. In some patients, however, there is a true aversion to baths, and in such they should not be tried.

Of drugs in a general way he condemns the use of iron. In some cases, especially in the young and those with arrested development, he states that he has obtained magnificent results with simple salt rubs or frictions. He has experimented extensively with acetate of lead, but finds it of little use except in pellagra of the aged, in those who suffer acute articular pain, in cases of incipient paresis, and in cases of general tremor. The dosage used was 0.01 to 0.05 gram in 300 c.c. of water. In typhoid pellagra he tried numerous remedies without avail.

Finally in his search for a remedy (through some reports of Coletti and Perugini) he got the idea of using arsenic, and he says, after experience with the drug, that the results exceeded by far his expectations. He does not seem to regard arsenic as a true specific for pellagra and admits that it does not cure all cases, but he thinks it is a very valuable remedy, and that it acts in a certain sense as an antidote for the toxins of spoiled maize, to which he attributes the disease. As an antidote, he compares it to the action of opium in alcoholism and mercury and the iodides in syphilis. Sodium chloride he seems to think has probably an equally powerful effect, but a very much more restricted field.

He uses arsenic in the form of Fowler's solution in dosage of 5, 10, 15, 20, and 30 drops, or in the form of pure arsenous acid (arsenic trioxide) dissolved in slightly alcoholised water, in doses of one-fortieth to one-twentieth milligram, increasing, according to tolerance, up to 0.001, 0.002, or 0.003 grams and very rarely even to 0.01 grams. The administration of the drug is suspended for a few days from time to time. He cautions against certain dangers in its use, however, and mentions as dangerous symptoms the appearance around the neck of an herpetic eruption, profuse salivation, anorexia, vomiting, diarrhea, palpitation of the heart, syncope, burning in the pharynx and stomach, headache, great muscular weakness, and bronchitis.

He thinks certain types are especially helped by the administration of arsenic, and that certain others receive no benefit, as follows:

*Benefited.*—Cases with marked marasmus; cases with incipient paresis; cases with sitophobia (gastralgic type); cases with vague mania but not systematised delirium; cases in the aged, if not at the verge of decrepitude.

*Not benefited.*—Cases in the young and in infants; cases well nourished and robust; cases with systematised delirium; cases with mental alienation of twenty to thirty years' duration; cases having lobar pneumonia, tuberculosis, albuminuria, or severe vertigo.

In cases of grave vertigo he sometimes uses the tincture of cocculus orientalis in doses of 3 to 5 drops daily, progressing slowly to 30 drops a day. Among systematic remedies he uses opium in certain mental states and calomel and bismuth for the diarrhea.

Rest is, of course, very important in acute manifestations, especially if accompanied by fever. The diet should be highly nutritious and abundant, including meats. If diarrhea is too free and the stools contain undigested material, it must be regulated accordingly. The diarrhea, however, is probably trophic and not inflammatory in nature, so that food is not contraindicated, as in many intestinal disturbances, and the patient needs all the nourishment possible. Change of climate, if possible, may be very advisable, especially to colder latitudes. Hydrotherapy is undoubtedly a valuable aid. Saline infusions may at times be of service. During the warm season avoidance of the sun's direct rays may prevent a bad erythema. Cleanliness and good nursing are, of course, to be desired.

Symptomatic remedies must be used as needed. For insomnia some of the well-known hypnotics; for the erythema, if dry, oily applications or possibly tincture of iodine; if moist, a dressing of 1 per cent. aqueous solution of picric acid is valuable at times, or other similar applications may be tried. Diarrhea must be met with the usual remedies; salicylate of bismuth has been highly recommended, and opium may prove of value. Pain, which is fortunately not very common or severe, may at times require morphine.

Complications, such as malaria, syphilis, and intestinal parasites, should receive prompt attention with approprite remedies. If much anemia be present, many good observers think a bland preparation of iron is indicated. Mercury, except in cases complicated by syphilis, seems valueless. Following Wright's work on the succinamide of mercury in tuberculosis, Babcock and I tried this remedy in several cases, but achieved no results except in syphilitic cases. The drug proved quite irritating locally.

*Use of the newer arsenical compounds.*—The more or less recent introduction of certain new arsenical compounds seemed, in the light of Lombroso's work, to offer a better therapy for pellagra. Atoxyl, first used, I think, by Babes, has been given a trial by several and with very discordant reports as to results. Of these preparations atoxyl and soamin are the only ones which have been used, so far as I am aware. Arsacetin is another important member of this group.

A few words on these drugs and their method of use may not be inappropriate. Atoxyl and soamin are both trade names and are forms of sodium arsenilate, containing, respectively, about 26 per cent. and 22 per cent. of arsenic. They are sold in the form of the salt itself and in the form of hypodermic tablets.

Sodium arsanilate is prepared by condensing aniline and arsenic acid, eliminating water and isolating the arsanilic acids. The sodium salt is prepared by the usual methods.

It occurs as white, odorless crystals soluble in 5 or 6 parts of water and more soluble in warm water.

*Action.*—The arsenic of the arsanilic acid is liberated very slowly in the system, thus producing the ordinary therapeutic effects of arsenic with the advantage of a more continuous and less toxic action and less irritation. Toxic effects from excessive doses have been frequently noted although the toxicity is stated to be about one-fortieth of that of arsenic trioxide. The use in large doses has occasionally resulted in blindness from degeneration of the optic nerve.

*Dose.*—0.02 to 0.2 grams (1-3 to 3 grains) hypodermically, every other day, gradually increasing if necessary until the single dose reaches 0.65 grams (10 grains) and until a total of 6.5 grams (100 grains) have been given. The drug should not be given by mouth, as it is decomposed by the acid of the stomach and toxic symptoms may result.

Arsacetin is sodium acetyl arsanilate. Its action is the same as sodium arsanilate. It is much more soluble and withstands heating so that its solutions may be sterilised. The dose is, hypodermically, 0.1 gram (1½ grains) to 0.5 gram (7½ grains), internally 0.05 gram (¾ grain) three or four times daily. If energetic action is required, two injections a week of 0.6 gram (9 grains) each, given on successive days, should be continued till 20 injections have been given. (This brief account of these remedies is abstracted from *Jour. Am. Med. Assn.* LII, No. 26, p. 2106.)

Koch in his extensive experience with atoxyl in trypanosomiasis, after getting several cases of blindness, concluded that the safest and most efficient dosage hypodermically was 0.5 gram on each of two succeeding days, and with intervals of ten days between; this double treatment is repeated for many months. By mouth Koch found that a dose of 0.5 gram is insufficient, while larger doses produced toxic symptoms, and he had no success with the drug given in this way. (*Terry, Arch. Int. Med.* III, 2.)

About two years ago Babes reported his experience with atoxyl in Roumania, and spoke very highly of the use of it in pellagra. Warnock, of the insane asylum at Cairo, Egypt, in his report for 1907, being somewhat enthusiastic over the Roumanian report, gave the remedy a trial, and was much pleased with his early results. In his report for 1908, however, his conclusion is, "It may be said that the value of atoxyl in the treatment of advanced stages of pellagra such as are met with in this asylum has not been demonstrated," and he adds that he can not confirm the Roumanian experience with the drug.

Babcock, at the State Insane Asylum, at Columbia, S. C., who has used both atoxyl and soamin extensively, has stated in a recent unpublished paper that he has not observed any permanent benefit from treatment by either of these preparations. He thinks, however, Fowler's solution is a remedy of importance, especially in non-asylum cases, and advocates, in selected cases, a further trial of atoxyl and soamin.

Babcock uses atoxyl and soamin almost exclusively by the intramuscular method. They have not proved irritating when sterile solutions were used and antiseptic precautions observed. The usual dosage is from about 0.2 to 0.5 gram every other day for two or three doses, and then a rest for about ten days.

Wood, of Wilmington, N. C. (*Char. Med. Jour.* LX, 2), speaks disparagingly of atoxyl in his experience.

In my own experience, atoxyl and soamin have proved of little value, but I am as yet not willing to discard them as entirely useless. Fowler's solution seems beneficial in some cases. Donovan's solution has been tried also, but I have had no experience with it myself.

Quite recently Babes, with others, has advocated the use of atoxyl and arsenic trioxide combined (*Berl. Klin. Wochenschrift,* February 8, 1909), and they report brilliant results. The method is as follows: atoxyl, 0.5 gram hypodermically, externally on the sound skin, 5 grams of an ointment of arsenic trioxide (1 to 50), and internally a pill of arsenic trioxide (0.001 to 0.002 gram) thrice daily. I have seen this treatment given a limited trial at Columbia, S. C., without any benefit.

*Serum treatment and transfusion of blood.*—A word or so on serum treatment and transfusion of blood. There is a good deal of evidence tending to show that specific antibodies are developed in the blood of pellagrins, and the serum of cured cases has been successfully used in the treatment of typhoid pellagra (Antonini and Mariani—Contributo allo studio della sieroterapia nella pellagra, Bergamo, 1904). Babes and Sion (loc. cit.) have even expressed the confident hope of producing from the horse an efficient antiserum, but this has not yet been realised.

Working at the insane asylum at Columbia, I have attempted to treat two cases with blood serum taken from cured pellagrins. One case died of pneumonia soon after treatment was begun, the other seemed to improve for a while, but is now much emaciated and is not expected to recover. I could not secure properly cured cases for obtaining the serum, and this may account to some extent for so poor a result.

Cole, of Mobile (*So. Med. Jour.*, April, 1909, 631-638), reports a case cured by transfusion of blood from a cured pellagrin (after Crile's method). He has recently tried this in other cases and reports good results. It seems to me possible that the blood from any healthy individual might have a similar beneficial result.

Finally, it may be said that we have no specific for the disease, and that the remedies used have often proved disappointing; but a cheerful optimism, with the judicious use of the means at our command, will at times produce surprisingly good results and is certainly far preferable to an inert pessimism.

Before concluding, I desire especially to express my indebtedness to Dr. J. W. Babcock, whose wide clinical experience with pellagra has rendered his information and advice of great value.

